# Effect of renal function on pemetrexed-induced haematotoxicity

**DOI:** 10.1007/s00280-016-3078-7

**Published:** 2016-06-10

**Authors:** Yosuke Ando, Takahiro Hayashi, Moeko Ujita, Sumie Murai, Hideki Ohta, Kaori Ito, Teppei Yamaguchi, Minori Funatsu, Yoshiaki Ikeda, Kazuyoshi Imaizumi, Kenji Kawada, Kimio Yasuda, Shigeki Yamada

**Affiliations:** Department of Clinical Pharmacy, School of Medicine, Fujita Health University, 1-98 Dengakugakubo, Kutsukake-cho, Toyoake, 470-1192 Japan; College of Pharmacy, Kinjo Gakuin University, 2-1723 Omori, Moriyama, Nagoya, 463-8521 Japan; Department of Hematology, School of Medicine, Fujita Health University, 1-98 Dengakugakubo, Kutsukake-cho, Toyoake, 470-1192 Japan; Department of Respiratory Medicine, School of Medicine, Fujita Health University, 1-98 Dengakugakubo, Kutsukake-cho, Toyoake, 470-1192 Japan; Department of Medical Oncology, School of Medicine, Fujita Health University, 1-98 Dengakugakubo, Kutsukake-cho, Toyoake, 470-1192 Japan

**Keywords:** Pemetrexed, Renal dysfunction, Thrombocytopaenia, Non-small cell lung cancer, Malignant pleural mesothelioma

## Abstract

**Purpose:**

Pemetrexed (PEM) is an anticancer agent used for the treatment of non-small cell lung cancer, malignant pleural mesothelioma and thymoma. Reportedly, PEM has higher efficacy and safety when used in combination with platinum-based agents. However, there are only few reports on the safety of PEM in patients with an eGFR of ≤45 mL/min. We examined the effect of renal function on the safety of regimens containing PEM.

**Methods:**

We retrospectively reviewed 221 patients with lung cancer, malignant pleural mesothelioma or thymoma who received treatment with a PEM-containing regimen between 2009 and 2014. Subgroup analyses were performed on the basis of pre-treatment renal function: group A [creatinine clearance (CLcr), <45 mL/min]; group B (CLcr, 45–80 mL/min); and group C (CLcr, ≥80 mL/min). For the purpose of this analysis, the lowest documented blood cell counts and haemoglobin levels, the highest levels of serum creatinine, aspartate aminotransferase, alanine aminotransferase and CLcr from the time of initial administration up to prior to the start of second administration were considered.

**Results:**

Groups A, B and C had 8, 123 and 90 patients, respectively. The incidence of grade 2 thrombocytopaenia was significantly higher in group A as compared to that in groups B (*P* < 0.01) and C (*P* < 0.05). On multivariate analysis, only a CLcr of <45 mL/min was an independent risk factor for thrombocytopaenia of ≥grade 2.

**Conclusion:**

When administering a PEM-containing regimen, thrombocytopaenia of ≥grade 2 is more likely to develop in patients with a CLcr of <45 mL/min.

## Introduction

The advent of new drugs and knowledge has revolutionised the treatment of lung cancer. Using epidermal growth factor receptor (EGFR) tyrosine kinase inhibitors for EGFR gene mutation-positive lung cancer [1–5] and anaplastic lymphoma kinase (ALK) inhibitors for echinoderm microtubule-associated protein-like 4-ALK translocation-positive lung cancer [6, 7] have improved overall survival (OS) in these patients. First-line treatment for stage IV lung cancer not associated with genetic mutations includes the combination of third-generation cytocidal antineoplastic preparations, such as irinotecan (CPT-11), paclitaxel (PTX), gemcitabine (GEM) and vinorelbine (VNR), with platinum-based preparations such as cisplatin (CDDP) and carboplatin (CBDCA) [8]. The specific regimens include CPT-11 + CDDP (IP) [9], PTX + CBDCA (TC) [10, 11], GEM + CDDP (GP) [12] and VNR + CDDP (NP) [12]. In the four-arm cooperative study [12], these four regimens were comparable in terms of therapeutic efficacy. However, much variability was observed with respect to the side effects. In particular, GP therapy was associated with a lower incidence of neutropaenia of ≥grade 3 and febrile neutropaenia (FN) when compared with the other regimens. Moreover, no treatment-related fatality was observed in patients who received GP. The GP regimen has since been considered the safest regimen among the four regimens. Furthermore, in the JMDB study [13] that compared pemetrexed (PEM) + CDDP [treatment for non-small cell lung cancer (NSCLC)] with GP therapy, the former was associated with significantly longer OS in patients with non-squamous NSCLC. Moreover, PEM + CDDP therapy was associated with a significantly lower incidence of neutropaenia, thrombocytopaenia of ≥grade 3 and FN, as compared to that associated with GP therapy. Based on these results, the National Comprehensive Cancer Network guidelines recommended PEM + CDDP therapy as the regimen with the highest efficacy and safety for EGFR mutation-negative and ALK translocation-negative stage IV non-squamous NSCLC [8].

However, in a phase I study [14], PEM (500 mg/m^2^) was shown to be well tolerated in patients with a glomerular filtration rate (GFR) >40 mL/min. Subsequently, in the JMDB study, PEM was administered to patients with a GFR of >45 mL/min, while those with a GFR of ≤40 mL/min continued to be excluded. Owing to the lack of definitive evidence on the efficacy and safety of PEM in the latter category of patients, PEM therapy is not recommended for such patients.

PEM plasma clearance is known to correlate with renal function [14]. Furthermore, patients with a GFR of ≤45 mL/min were shown to have a high incidence of PEM-induced severe neutropaenia [15, 16]. However, as shown in the JMDB study, PEM + CDDP was shown to have the highest efficacy and safety in patients with NSCLC. Moreover, other recent studies have also suggested the efficacy of PEM monotherapy for NSCLC [17, 18].

Therefore, it is necessary to examine tolerance in patients with a GFR of ≤45 mL/min for lung cancer treatment. Currently, the safety of PEM-containing regimens has not been established for patients with a GFR of ≤45 mL/min, and as evaluation by prospective clinical trials is not ethically permitted, it is relevant first to perform a preliminary retrospective study.

Therefore, we retrospectively analysed data on patients who had received a PEM-containing regimen at our hospital. The objective was to investigate the effect of renal function on the incidence of PEM-induced side effects.

## Materials and methods

### Subjects

Patients treated with a PEM-containing regimen [PEM + CDDP; PEM + CDDP + bevacizumab (Bev); PEM + CBDCA; PEM + CBDCA + Bev; PEM + Bev; and PEM monotherapy] for lung cancer, malignant pleural mesothelioma or thymoma at Fujita Health University Hospital between 1 September 2009 and 31 August 2014 were enroled in the study. Patients with renal dysfunction induced by other agents and patients for whom renal function could not be assessed prior to the start of PEM treatment were excluded.

### Investigations

Data were accessed from the electronic medical records available at Fujita Health University Hospital. Renal function investigated prior to the start of treatment was used for the categorisation of patients according to renal function: group A [patients with creatinine clearance (CLcr) of <45 mL/min], group B (CLcr 45–80 mL/min) and group C (CLcr ≥80 mL/min). CLcr was calculated from serum creatinine levels using the Cockcroft–Gault equation [19]. The standard method to measure serum creatinine levels is the Jaffe method. However, we used the enzyme method, which is commonly used in Japan, and calculated serum creatinine levels by adding 0.2 to the actual measured values [20, 21].

Data on baseline variables such as age, sex, body surface area and the presence or absence of cancer metastasis and invasion prior to the start of treatment were obtained. Data on the following treatment parameters were obtained: initial dose of pemetrexed (mg/m^2^), initial dose of cisplatin (mg/m^2^), initial dose of carboplatin (mg/m^2^), co-administration of oral folic acid tablets and vitamin B_12_ (because they may help reduce PEM-induced side effects [22–24]) and/or use of non-steroidal anti-inflammatory drugs (NSAIDs) (which can exacerbate PEM-induced side effects [25, 26]).

### Assessment

The lowest documented values of blood cell counts (leucocyte, neutrophil, red blood cell and platelet counts), haemoglobin and CLcr and the highest levels of serum creatinine, aspartate aminotransferase (AST) and alanine aminotransferase (ALT) were assessed on the basis of the National Cancer Institute—Common Terminology Criteria for Adverse Events version 4.0 (NCI-CTCAE version 4.0).

### Statistical analysis

Data on variables with normal distribution are expressed as mean ± standard deviation. Non-normally distributed variables are expressed as means with interquartile ranges. Between-group differences with respect to normally distributed variables (expressed as frequencies) were assessed using the one-way repeated measures analysis of variance (ANOVA) while non-normally distributed variables were compared using the Kruskal–Wallis test. Data expressed as percentages were assessed by Chi-square test. For multiple comparisons, Bonferroni correction was applied after performing a Chi-square test for two groups. Univariate analysis was performed to identify risk factors. Variables with a significance level of <20 % were included in the multivariate logistic regression model. Hosmer–Lemeshow statistical test was used to verify goodness of fit. Statistical analyses were performed using SPSS version 22.0 (IBM Corporation, Armonk, NY, USA). Between-group differences associated with a *P* value of <0.05 were considered statistically significant.

### Ethics

The present study was conducted in compliance with the ‘ethical guidelines for clinical research’. The study protocol was approved by the ‘Ethics committee for epidemiological and clinical research’ at our hospital.

## Results

### Patients

The study population consisted of 221 subjects [group A (*n* = 8), group B (*n* = 123) and group C (*n* = 90)]. All subjects were administered both oral folic acid tablets and vitamin B_12_. A statistically significant difference was observed between the three groups with respect to age, treatment regimen, pemetrexed dose and concurrent use of NSAIDs (Table 1).

**Table 1:** Patient background (before chemotherapy)

	Group A (*n* = 8)	Group B (*n* = 123)	Group C (*n* = 90)	*P* value
Age (years)	72.5 (70.5–75.3)	72.0 (67.0–76.0)	62.0 (57.0–68.8)^a,c^	<0.001 (Kruskal–Wallis test)
Sex (male, female)	5, 3	79, 44	67, 23	0.363 (*χ* ^2^ test)
CLcr (mL/min)	38.0 (33.7–41.3)	63.3 (55.4–73.3)^b^	94.3 (85.9–102.3)^b,c^	<0.001 (Kruskal–Wallis test)
Cancer classification				0.827 (*χ* ^2^ test)
Primary tumour	4	69	48	
Metastasis or infiltration tumour	4	54	42	
Chemotherapy regimen				0.026 (*χ* ^2^ test)
PEM monotherapy	1	26	17	
PEM + CDDP	0	36	41	
PEM + CDDP + Bev	0	2	1	
PEM + CBDCA	7	51	24	
PEM + CBDCA + Bev	0	8	7	
Dosage of PEM (mg/m^2^)	432.7 ± 66.7	488.3 ± 16.8^d^	485.1 ± 25.3^d^	<0.001 (one-way ANOVA)
NSAIDs	0	33	36	0.013 (*χ* ^2^ test)

*CLcr* creatinine clearance, *PEM* pemetrexed, *CDDP* cisplatin, *CBDCA* carboplatin, *NSAIDs* non-steroidal anti-inflammatory drugs

^a^ *P* < 0.05 versus group A (Steel–Dwass test)

^b^ *P* < 0.01 versus group A (Steel–Dwass test)

^c^ *P* < 0.01 versus group B (Steel–Dwass test)

^d^ *P* < 0.01 versus group A (Tukey test)

### Regimen

Statistically significant differences were observed between the three groups in terms of the usage rate of the CDDP-containing regimen. On multiple comparisons, the usage rate for group C was significantly higher than that for group A (*P* = 0.032) and tended to be higher than that for group B (*P* = 0.056). No difference was observed between groups A and B (*P* = 0.21). Furthermore, between-group differences were observed with respect to the usage rate of CBDCA-containing regimen. On multiple comparisons, the usage rate for group A was significantly higher than that for group C (*P* = 0.0095) and tended to be higher than that for group B (*P* = 0.090). No difference was observed between groups B and C (*P* = 0.14). There was no difference between the three groups in terms of the usage rate of Bev-containing regimen (Table 2).

**Table 2:** Combination chemotherapy regimens containing pemetrexed

	Group A (*n* = 8)	Group B (*n* = 123)	Group C (*n* = 90)	*P* value
CDDP plus PEM				
No. of patient (%)	0 (0 %)	38 (30.9 %)	42 (46.7 %)	0.007 (*χ* ^2^ test)
Dosage (mg/m^2^)	–	73.2 (71.9–74.0)	73.3 (72.4–74.4)	0.64 (Mann–Whitney *U* test)
CBDCA plus PEM				
No. of patient (%)	7 (87.5 %)	59 (48.0 %)	31 (34.4 %)	0.008 (*χ* ^2^ test)
Target AUC	4.68 ± 1.23	4.93 ± 0.77	5.15 ± 0.80	0.53 (one-way ANOVA)
Bev plus PEM				
No. of patient (%)	0 (0 %)	10 (8.1 %)	8 (8.9 %)	0.56 (*χ* ^2^ test)

*PEM* pemetrexed, *CDDP* cisplatin, *CBDCA* carboplatin, *AUC* area under the concentration–time curve, *Bev* bevacizumab

### Safety

When assessing the onset of adverse events in each group by grade, the incidence of grade 1 thrombocytopaenia was higher in group B as compared to that in group C, while for grade 2, the incidence was higher in group A as compared to that in groups B and C. No significant association of severe adverse events (≥grade 3) with leucocyte, neutrophil and red blood cell counts, haemoglobin levels (Table 3) and elevated AST and ALT was observed between the three groups (Table 4).

**Table 3:** Incidence of haematotoxicity by grade and study group

	Group A (*n* = 8)	Group B (*n* = 123)	Group C (*n* = 90)	*P* value (*χ* ^2^ test)
Leukopaenia				
Grade 1	0 (0 %)	10 (8.1 %)	13 (14.4 %)	0.219
Grade 2	2 (25.0 %)	36 (29.3 %)	19 (21.1 %)	0.362
Grade 3	2 (25.0 %)	15 (12.2 %)	10 (11.1 %)	0.510
Grade 4	0 (0 %)	0 (0 %)	2 (2.2 %)	0.237
Neutropaenia				
Grade 1	1 (12.5 %)	21 (17.1 %)	18 (20.0 %)	0.816
Grade 2	2 (25.0 %)	29 (23.6 %)	21 (23.3 %)	0.988
Grade 3	2 (25.0 %)	18 (14.6 %)	11 (12.2 %)	0.567
Grade 4	1 (12.5 %)	7 (5.7 %)	5 (5.6 %)	0.718
Anaemia				
Grade 1	4 (50.0 %)	71 (57.7 %)	48 (53.3 %)	0.831
Grade 2	1 (12.5 %)	20 (16.3 %)	20 (22.2 %)	0.528
Grade 3	0 (0 %)	5 (4.1 %)	7 (7.8 %)	0.409
Grade 4	0 (0 %)	0 (0 %)	0 (0 %)	–
Thrombocytopaenia				
Grade 1	3 (37.5 %)	68 (55.3 %)	35 (38.9 %)^c^	0.037
Grade 2	4 (50.0 %)	6 (4.9 %)^a^	7 (7.8 %)^b^	<0.001
Grade 3	1 (12.5 %)	8 (6.5 %)	2 (2.2 %)	0.214
Grade 4	0 (0 %)	4 (3.3 %)	3 (3.3 %)	0.873

*χ* ^2^ test with Bonferroni correction

^a^ *P* < 0.01 versus group A

^b^ *P* < 0.05 versus group A

^c^ *P* < 0.05 versus group B

**Table 4:** Effect of chemotherapy on liver function

	Group A (*n* = 8)	Group B (*n* = 123)	Group C (*n* = 90)	*P* value (*χ* ^2^ test)
AST increased				
Grade 1	3 (37.5 %)	39 (31.7 %)	35 (38.9 %)	0.559
Grade 2	0 (0 %)	5 (4.1 %)	4 (4.4 %)	0.852
Grade 3	0 (0 %)	0 (0 %)	1 (1.1 %)	0.491
Grade 4	0 (0 %)	0 (0 %)	0 (0 %)	–
ALT increased				
Grade 1	4 (50.0 %)	67 (54.5 %)	53 (58.9 %)	0.702
Grade 2	1 (12.5 %)	4 (3.3 %)	8 (8.9 %)	0.172
Grade 3	0 (0 %)	4 (3.3 %)	6 (6.7 %)	0.423
Grade 4	0 (0 %)	0 (0 %)	0 (0 %)	–

*AST* aspartate aminotransferase, *ALT* alanine aminotransferase

### Risk factors

We examined potential risk factors for thrombocytopaenia of ≥grade 2. Univariate analysis revealed a significant difference for the three following factors: CLcr of <45 mL/min, CDDP administration and CBDCA administration. In contrast, for other factors such as age, PEM dosage and NSAIDs administration, no significant difference was observed. On multivariate analysis of the three factors that exhibited a significance level of <20 % on univariate analysis, only a CLcr of <45 mL/min was an independent risk factor. The administration of platinum-based preparations was not found to be a risk factor (Table 5). Among the eight patients with a CLcr of <45 mL/min, none of the five patients who developed thrombocytopaenia of ≥grade 2 exhibited a decrease in CLcr of >10 mL/min over that at the time of initiation of the second course of treatment.

**Table 5:** Risk factors for thrombocytopaenia of ≥grade 2

	Univariate analysis		Multivariate analysis	
	Odds ratio (95 % CI)	*P* value	Odds ratio (95 % CI)	*P* value
Age (years)	1.02 (0.98–1.06)	0.26		
Male	1.01 (0.47–2.21)	0.97		
CLcr < 45 mL/min	10.17 (2.31–44.77)	0.002	6.40 (1.41–29.10)	0.016
Metastasis or infiltration tumour	1.54 (0.75–3.18)	0.24		
CDDP	0.25 (0.09–0.67)	0.006	0.56 (0.15–2.08)	0.39
CBDCA	3.96 (1.80–8.73)	0.001	2.47 (0.86–7.09)	0.093
Bev	0.29 (0.04–2.27)	0.24		
Dosage of PEM (mg/m^2^)	1.00 (0.99–1.02)	0.93		
NSAIDs	0.85 (0.39–1.89)	0.70		

Predictive ability of final model quantified using the Hosmer–Lemeshow statistical test for goodness of fit; *P* = 0.756

*CI* confidence interval, *CLcr* creatinine clearance; *CDDP* cisplatin, *CBDCA* carboplatin, *Bev* bevacizumab, *PEM* pemetrexed, *NSAIDs* non-steroidal anti-inflammatory drug

## Discussion

The safety of PEM-containing regimens in patients with a CLcr of <45 mL/min has yet to be established. In this study, administration of a PEM-containing regimen to patients with a CLcr of <45 mL/min was associated with a significantly higher incidence of thrombocytopaenia of ≥grade 2. Furthermore, the propensity of CBDCA to cause thrombocytopaenia is well known [27–29], and upon identification of risk factors for thrombocytopaenia of ≥grade 2, we found a CLcr of <45 mL/min to be an independent risk factor. In other words, CBDCA and other factors appeared to have had little effect. Moreover, although the dosage was low in group A, the onset of thrombocytopaenia of ≥grade 2 appeared not to be affected by PEM dosage. This suggests that renal function is a stronger correlate of the onset of thrombocytopaenia in patients receiving PEM therapy. The incidence of other types of haematotoxicity (as evidenced by haematological results) and liver dysfunction also appeared to be unaffected by renal function.

On subgroup analysis, none of the patients with a CLcr of <45 mL/min who developed thrombocytopaenia of ≥grade 2 showed a decrease in CLcr by ≥10 mL/min by the start of the second course. This supports the notion that a further decline in renal function did not affect the incidence of thrombocytopaenia in the present study. Fall in platelet counts generally occurs 7–10 days after the initiation of anticancer therapy, and the period of platelet decrease is considered to be approximately 14 days. In the present study, the mean platelet nadir period was 13 days, which is consistent with earlier reports. Therefore, it may be inferred that the occurrence of thrombocytopaenia of ≥grade 2 in patients with compromised renal function does not necessarily imply a specific mechanism, but only the incidence is increased.

Furthermore, in the present study, the incidence of haematotoxicity in patients with a CLcr of <45 mL/min included neutropaenia of ≥grade 3 (37.5 %) and thrombocytopaenia of ≥grade 3 (12.5 %). A breakdown of treatment methods revealed that PEM + CBDCA therapy was used in 7 out of 8 patients. This result was not remarkably higher than those reported by Schuette et al. [30] who evaluated PEM + CBDCA therapy (neutropaenia, 26.2 %; thrombocytopaenia, 16.9 %). Therefore, it is inferred that the incidence of haematotoxicity in patients with a CLcr of <45 mL/min was within the permissible range.

With regard to patient background, a significant difference was observed between the groups in terms of age, type of chemotherapy regimen and use of NSAIDs. With regard to age, as groups were divided according to renal function, the fact that more elderly patients were included in groups A and B is consistent with earlier reports [31]. In group A, a CBDCA-combined regimen was most often used. In group C, a CDDP-combined regimen was most often used, which was assumed to be affected by the following facts: the CDDP dosage needs to be adjusted according to renal function [32] and because CDDP therapy has been found to cause renal dysfunction [33], it is difficult to use CDDP in patients who have pre-existing renal impairment prior to the start of chemotherapy. Moreover, multivariate analysis revealed that the three factors (age, type of chemotherapy regimen and use of NSAIDs) had little effect on the onset of thrombocytopaenia of ≥grade 2.

Our results suggest the need for close monitoring of platelet counts in patients with a CLcr of <45 mL/min undergoing treatment with PEM-containing regimens.

The retrospective nature of this study is a key limitation. Assessment of non-haematotoxicities that could not be evaluated on the basis of laboratory results should be further studied. PEM therapy is not currently recommended in patients with a CLcr of <45 mL/min in Japan, which explains the small sample size of eight patients in group A. This is likely to have introduced a bias with respect to patient characteristics (Table 1). However, multivariate analysis ruled out all risk factors other than renal function. Therefore, we believe that the results of the present study are useful. Our results along with those of other retrospective studies may help to determine the optimal dosage levels for patients with compromised renal function and may help improve the safety profile of PEM-containing regimens.

Considering the fact that renal function declines with age [31], along with a relatively higher incidence of NSCLC in elderly patients aged >70 years [34], it is imperative to establish the safety of PEM-containing regimens for patients with renal dysfunction. When using PEM-containing regimens in patients with renal dysfunction corresponding to a CLcr of <45 mL/min, attention should be given to the potential onset of thrombocytopaenia.
